# Thyrotoxic Muscle Paralysis as a Rare Cause of Reversible Muscle Weakness: A Case Report

**DOI:** 10.7759/cureus.10634

**Published:** 2020-09-24

**Authors:** Qasim Z Iqbal, Zeeshan Zia, Muhammad Niazi, Saud Bin Abdul Sattar, Shahed Quyyumi

**Affiliations:** 1 Internal Medicine, Northwell Health, New York, USA; 2 Endocrinology, Diabetes and Metabolism, Northwell Health, New York, USA

**Keywords:** reversible cause of muscle weakness, hyperthyroidism, thyrotoxicity, hypokalemia

## Abstract

Thyrotoxic periodic paralysis is a clinical condition characterized by muscle weakness in patients with underlying hyperthyroidism. It is usually more commonly seen in patients of Asian origin and has a predisposition for the male population (unlike other thyroid disorders which commonly affect the female population). Findings are more overt in patients who have subclinical hyperthyroidism and there is a risk of them remaining untreated. The symptoms can range from mild muscle weakness to total paralysis. The muscles affected predominantly are the proximal and lower extremities group of muscles. Thyrotoxic muscle paralysis can be precipitated by hyperinsulinemic states such as after heavy meals, physical exertion, obesity, stress, and certain medications like high-dose steroids, antiretrovirals, and interferon therapy. The acute intervention usually revolves around the replenishment of patient's potassium stores followed by maintenance therapy with anti-thyroid medications. We present a case of a Chinese adolescent who presented to us with lower muscle weakness and underlying subclinical hyperthyroidism. It's important for clinicians to be familiar with this disease entity and include it in their differentials whenever a patient with muscle weakness presents. The treatment is simple and can result in rapid improvement of symptoms and should be initiated quickly.

## Introduction

Thyrotoxic periodic paralysis is a rare disorder that causes occasional episodes of muscle weakness. This is associated with a lower than normal levels of potassium (hypokalemia) in the blood and a clinical state of the hyperactive thyroid gland (hyperthyroidism). There is usually a rapid improvement in symptoms if these patients are diagnosed correctly, and thyroid directed treatment is started. Clinicians should be aware of it because of the wide overlap in symptoms of patients with thyrotoxic periodic paralysis and periodic paralysis. Among various differentials of flaccid paralysis, it is unique because of its remitting nature, and if diagnosed correctly, accurate treatment can result in significant clinical improvement within a very short period of time. Therefore the physicians need to appreciate this diagnosis while seeing patients with flaccid paralysis.

## Case presentation

A 30-year-old Asian male, who was recently diagnosed with subclinical hyperthyroidism but not started on treatment since he did not have symptoms, presented to the Emergency Department with the complaint of worsening muscle weakness. The muscle weakness started around three weeks ago. This weakness was gradual in onset, and the patient reported it to be worse at the end of the day. It was primarily located bilaterally around the thighs, which made it difficult for the patient to stand. The patient said that the morning before he came to the hospital, he felt too weak to move his legs even, and he was not able to stand properly. On initial examination in the emergency department, he was found to have weakness in both his upper (4/5 in muscle strength) and lower extremities (3/5 in muscle strength). The patient’s reflexes were decreased throughout all extremities. There were no other neurologic deficits present in the initial assessment of the patient. Laboratory work was done, and the initial laboratory studies showed that the patient was extremely hypokalemic (1.9 meq/L). The patient immediately received potassium intravenously, after which there was a significant improvement in his symptoms.

The very next day, the patient’s potassium levels improved significantly to 4.9, and simultaneously there was also a major improvement in his muscle strength that by the second day was 5/5 in all the extremities. The initial thyroid function tests that came out the following day showed hyperthyroidism with an elevated total T3 (273 ng/dL) and T4 (10.3 mcg/dL) along with a suppressed thyroid-stimulating hormone (TSH <0.05 mIU/mL). The endocrinology team was consulted and the patient was diagnosed with hypokalemic periodic paralysis secondary to hyperthyroidism. The patient was started on non-selective beta-blockers and anti-thyroid medications and later discharged home after monitoring in the intensive care unit (ICU) for two days.

## Discussion

Considering the various differentials of flaccid paralysis, periodic paralysis is unique because of its remitting nature. There is only one distinct entity of periodic paralysis associated with hyperthyroidism, which is thyrotoxic periodic paralysis. It is a clinical condition seen more commonly in Asian patients and presents with weakness of muscles. The weakness is usually more severe in the lower extremities, mainly a predisposition for proximal muscles [1, 2].

It usually accompanies hyperthyroid symptoms such as tachycardia, anxiety, palpitations, exophthalmos, heat intolerance, tremors, and weight loss [3, 4]. The disease severity can vary from mild weakness to quadriplegia to even total paralysis. In these patients, there is usually no family history of muscle paralysis. The bulbar, respiratory, and ocular muscles are, in most cases, spared. The clinical case can be tricky if the patient has subclinical hyperthyroidism, and the presenting complaint is only paralysis or decreased muscle strength in the limbs. There are very few reported cases of thyrotoxic periodic paralysis with subclinical hyperthyroidism [5].

The pathophysiology behind the paralysis is explained by hyperthyroidism leading to a hyper-adrenergic state that activates beta receptors ramping up the ATPase activity, causing pumping of potassium intracellularly. This results in hypokalemia, without any renal or gastrointestinal loss; hence the total body potassium stays normal. The hyperthyroid patients are known to have a high beta-adrenergic activity that pumps potassium inside the cells, causing hypokalemia that mainly results in muscle weakness. Insulin resistance and alkalosis also cause hypokalemia by the same mechanism. It is important to mention that the total potassium levels inside the body usually remain the same. There is usually no excessive gastrointestinal or urinary loss of potassium [6]. The severity of the disease is not found to be correlated with the degree of hyperthyroidism, even though the muscle paralysis is known to be resolved once the euthyroid state is achieved.

It is crucial to differentiate between familial hypokalemic paralysis and thyrotoxic periodic paralysis. The latter is more commonly seen in the third to fourth decades of life and males [7]. In thyrotoxic periodic paralysis, the treatment emphasizes on potassium supplementation (limited to < 60 meq/day to avoid rebound hyperkalemia) and starting the patient on non-selective beta-blocker and anti-thyroid treatment medications like methimazole and propylthiouracil [8].

## Conclusions

Flaccid paralysis is one of the unusual complications of hyperthyroidism. The purpose of this case report is to highlight the importance to consider thyrotoxic periodic paralysis among the differentials for patients coming to the emergency department with muscle weakness or paralysis. The clinicians should, furthermore, be aware of it because there is a wide overlap of symptoms among diseases presenting with muscle weakness. When diagnosed correctly, accurate treatment can result in significant clinical improvement within a very short period of time.
